# Annotated checklist of marine fishes from the Sanctuary of Bahía Chamela, Mexico with occurrence and biogeographic data

**DOI:** 10.3897/zookeys.554.6106

**Published:** 2016-01-18

**Authors:** Cristian Moisés Galván-Villa, Eduardo Ríos-Jara, Dafne Bastida-Izaguirre, Philip A. Hastings, Eduardo F. Balart

**Affiliations:** 1 Laboratorio de Ecosistemas Marinos y Acuicultura, Departamento de Ecología, Centro Universitario de Ciencias Biológicas y Agropecuarias, Universidad de Guadalajara. Zapopan, Jalisco, Mexico. 45110; 2 Marine Biology Research Division, Scripps Institution of Oceanography, University of California. San Diego, La Jolla, CA. 92093-0208; 3 Laboratorio de Necton y Ecología de Arrecifes, Centro de Investigaciones Biológicas del Noroeste, S.C. La Paz, Baja California Sur, Mexico. 23096

**Keywords:** Species richness, Ichthyofauna, eastern Pacific, systematic list, biogeographic affinity

## Abstract

An annotated checklist of marine fishes of the Sanctuary of Islands and Islets of Bahía Chamela in the central Mexican Pacific is presented. Records of fish species were obtained by different methods including visual census, sampling with anesthetics, fisherman-nets, and trawling with a biological dredge. Additional records were obtained from natural history collections and publications. The list comprises 196 species in 64 families and 141 genera. The Carangidae is the most speciose family with 11 species, followed by the Labridae with 10 and the Pomacentridae with nine. Fourteen species are endemic in Mexican Pacific waters, but none is restricted to Bahía Chamela. The most dominant species recorded during underwater surveys were *Epinephelus labriformis*, *Stegastes flavilatus*, and *Halichoeres dispilus*. Most species are of tropical affinities distributed throughout the tropical eastern Pacific (123), eastern Pacific (23), and Mexican Pacific (14). Other species are known from the eastern and Indo-Pacific regions (18), eastern Pacific and western Atlantic oceans (2), and some are circumtropical (9). A new record of the Gulf Brotula *Ogilbia ventralis* is provided for the Bahía Chamela and its geographical distribution is extended to Mexican central Pacific.

## Introduction

The study of fish diversity along the Mexican Pacific coasts started two centuries ago by naturalists and scientists who studied rich collections from some now-memorable expeditions (Gilbert 1890, Jordan et al. 1895, Breder 1926, 1927, 1928, 1936, Fowler 1944). Today the estimated number of recorded marine species along these coasts is 1,121, with the Gulf of California exhibiting the highest species richness (van der Heiden and Findley 1988, Hastings et al. 2010, Espinosa-Pérez 2014). However, there are still many areas and habitats (bays, estuaries, mangroves, reefs, littoral zones, deep-water realm) in the Mexican tropical Pacific where proper fish inventories are missing.

Fishes are an important marine group from an ecological and economic point of view. The destruction and pollution of many habitats and the overexploitation of fishes have affected marine ecosystems with the consequent loss of environmental services. For this reason, the implementation of Marine Protected Areas (MPAs) has begun to be a common practice in conservation and a useful fisheries management tool (Roberts et al. 2001, Edgar 2011). However, the design of an effective MPA requires information about the diversity of species inhabiting an area and its connectivity with other areas (Halpern and Warner 2003, Costello et al. 2010).

In the Mexican Pacific, there are some well-inventoried MPAs. For instance, there are well-documented checklists of fishes inhabiting Isla Guadalupe Biosphere Reserve (Reyes-Bonilla et al. 2010), an important area for the reproduction of the white shark off the Baja California peninsula. MPAs inside the Gulf of California include the Bahía de Los Ángeles Biosphere Reserve (Viesca-Lobatón et al. 2008, Mascareñas-Osorio et al. 2011), a seasonal sanctuary for the whale shark; Loreto Marine Park (Campos-Dávila et al. 2005, Rife et al. 2013); National Park Archipielago of Espíritu Santo (Aburto-Oropeza and Balart 2001, Arreola-Robles and Elorduy-Garay 2002, Rodríguez-Romero et al. 2005); Gulf of California Islands (Del Moral-Flores et al. 2013); Cabo Pulmo National Park (Alvarez-Filip et al. 2006), where sound management has restored the fish biomass (Aburto-Oropeza et al. 2011); Isla Isabel National Park (Galván-Villa et al. 2010); and Islas Marias Biosphere Reserve (Erisman et al. 2011). Others include the Archipielago de Revillagigedo Biosphere Reserve (Jordan and McGregor 1899, Castro-Aguirre and Balart 2002, Chávez-Comparán et al. 2010), Islas Marietas National Park (Solís-Gil and Jiménez-Quiroz 2004, García-Hernández et al. 2014), and Bahías de Huatulco National Park (Ramírez-Gutiérrez et al. 2007, López-Pérez et al. 2010, Juárez-Hernández et al. 2013) in the central and southern Mexican Pacific. However, many of the MPAs from the Mexican central Pacific are lacking inventories of marine fishes. One of these is the Sanctuary of Bahía Chamela located along the coast of Jalisco; it comprises eight islands and four islets dispersed along the bay.

The Sanctuary of Bahía Chamela was the first marine sanctuary in Mexico and has been protected since 2002 (Miranda et al. 2011). This sanctuary is home to species of restricted distribution and endemic fauna and flora. However, scarce information about fish diversity of the sanctuary is available. Only two previous lists of fishes of this bay are found reporting 59 and 80 species for the mainland coastline and for the two largest islands in the bay, respectively (Espinosa-Pérez et al. 2002, Galván-Villa 2015). In the current study, a comprehensive checklist of fish species from the Sanctuary of Bahía Chamela Islands has been compiled based on sampling work from 2007 to 2014, review of material from ichthyological collections, and critical analysis of selected references. A biogeographic and occurrence characterization of all species is also provided.

## Material and methods

**Figure 1.**
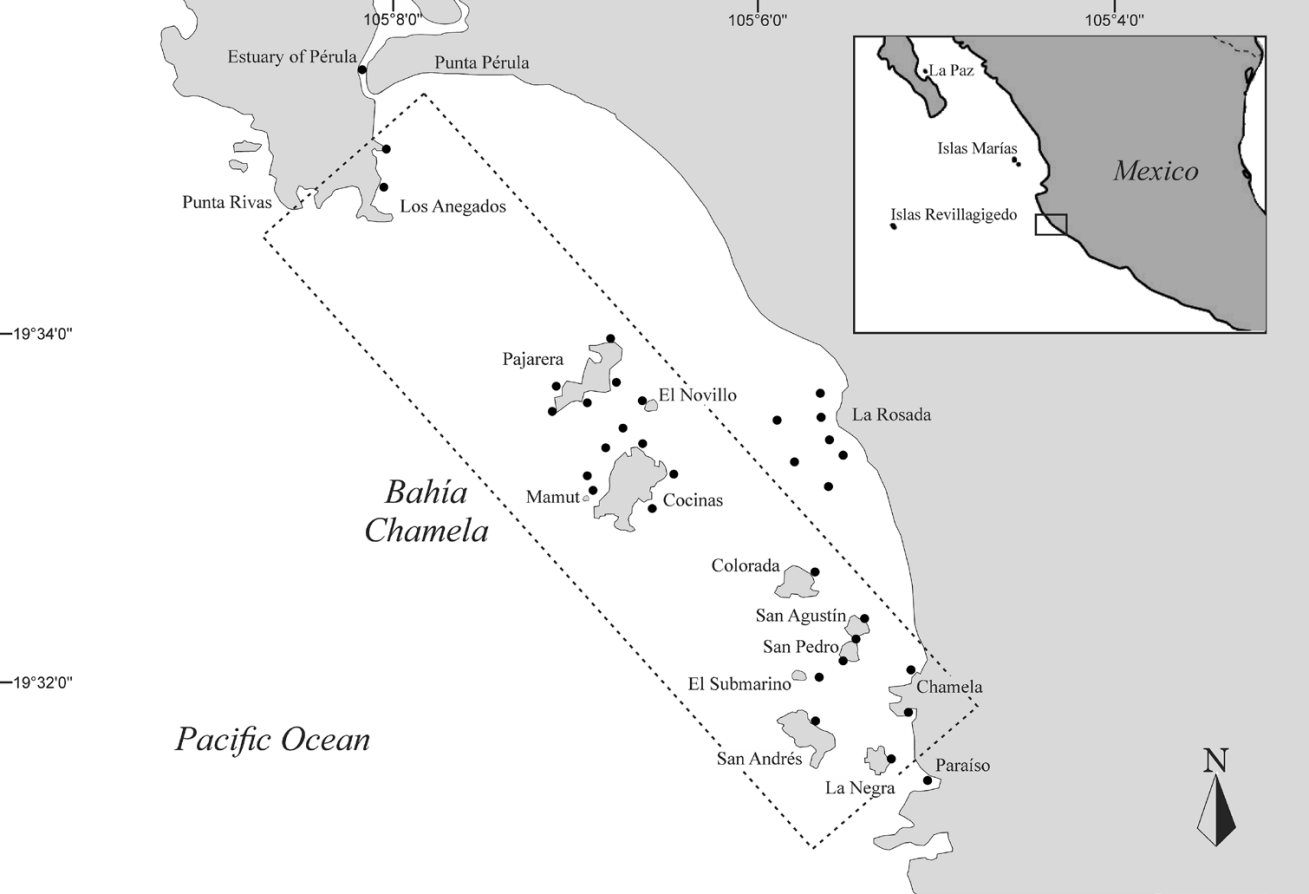
Location of Bahía Chamela, Jalisco, Mexico. Black dots show the location of the sampling sites in the bay. The dotted line indicates the limits of the Marine Protected Area.

*Study area.* The Bahía Chamela is located in the middle coastline area of Jalisco state on the central Mexican Pacific (19°32'N; 105°06'W) (Figure 1). The bay is located between two major oceanic systems: the Gulf of Tehuantepec and the Gulf of California. The extent of the bay is 28 km from Punta Chamela to Punta Rivas (south to north). The sanctuary includes eight islands called as Pajarera, Cocinas, Mamut, Colorada, San Pedro, San Agustín, San Andrés, and La Negra, and four islets as Los Anegados, El Novillo, La Mosca, and Submarino (CONANP 2008). All of these islands and islets are included in the Marine Priority Region No. 38 of sites for conservation of the National Commission for Knowledge and Use of Biodiversity of Mexico (CONABIO). The continental coast of the bay presents sandy beaches to the northern side and shallow plains and rocky beaches to the south. The islands and islets are of continental origin with similar age and composition throughout the region (possibly from the Cretaceous) (Schaaf 2002). The two larger islands have rocky and sandy beaches, while the smaller islands and the islets have rocky intertidal zones sometimes with vertical steep slopes. The depth of the bay varies between 10 and 25 m, decreasing dramatically in the proximity of the coastline and the islands.

*Sampling effort and data analysis.* Records of fish species were made by visual census and obtained from analyses of collection reports and materials and available publications. Records *in situ* were made using underwater visual census from 2007 to 2012 according to the technique described by English et al. (1994). Each transect covered an area of 100 m^2^ (50 m × 2 m) and was conducted by a single diver. Cryptic fishes and other specimens were collected from 2007 to 2015 with a 10% anaesthetic solution of clove oil diluted in ethanol, with a fisherman-net, and with a biological dredge. All collected specimens were deposited in the fish collection of the Laboratory of Marine Ecosystems and Aquaculture (LEMA-CPE), Centro Universitario de Ciencias Biológicas y Agropecuarias, Universidad de Guadalajara (Zapopan, Mexico), except specimen of *Chaenopsis* sp. that was deposited in the Marine Vertebrate Collection (SIO), Scripps Institution of Oceanography, University of California (San Diego, USA). Records obtained from publications included only those that were identified to species level and excluded any questionable records that we could not confirm as species known to occur in the Mexican Pacific.

The nomenclature for species level, family designations, and systematic were updated following Eschmeyer (2015). Distributions and biogeographic affinities for species are based on Thomson et al. (2000), Hastings and Springer (2009), Erisman et al. (2011), Mascareñas-Osorio et al. (2011), and Robertson and Allen (2015), using the following categories: CT = Circumtropical (distributed throughout the tropics of the world), EP = eastern Pacific (including tropical and temperate regions), EP+ATL = eastern Pacific and western Atlantic oceans (occurs in both oceans), EP+IP = eastern Pacific and Indo-Pacific regions (occurs in both regions), MEX = Mexican waters of the Pacific (including the Gulf of California and outer coast of Baja California), and TEP = tropical eastern Pacific (extends from south of Magdalena Bay, Baja California to Cabo Blanco in northern Peru, includes the Gulf of California and offshore islands as Revillagigedo, Clipperton, Cocos, Malpelo, and the Galápagos). For a description of the structure of fish assemblages, the species recorded between 2007 and 2012 through visual census were classified in five categories using the frequency of occurrence: D = Dominant (> 80% of census), A = Abundant (61-80%), C = Common (41-60%), U = Uncommon (21-40%), and R = Rare (< 21%).

## Results and discussion

*Species richness.* A list of 196 species, 141 genera, and 64 families of marine fishes from the Bahía Chamela is presented (Table 1). In comparison with previous studies (Espinoza-Pérez et al. 2002, Galván-Villa 2015), the richness of the bay increased in this study by more than 240% (by 117 species). The jacks (Carangidae) represent the most diverse family, with 11 species and 8 genera, followed by the wrasses (Labridae) with 10 species and 5 genera, and damselfishes (Pomacentridae) and grunts (Haemulidae) each with 9 species and 4 genera. Eighteen families are represented by only one species. No endemic species for Bahía Chamela were found but 14 endemic species for the Mexican Pacific are recorded here.

**Table 1.** Checklist of fishes from the Sanctuary of Bahía Chamela, Mexico. The list is arranged systematically by class, orders, and families according to Eschmeyer (2015). Record designation: V = visual record (2007–2009); C = collected specimen (2007-2015); SIO = records of the Scripps Institution of Oceanography; R1 = Espinoza-Pérez et al. (2002); R2 = Galván-Villa (2015). Frequency of occurrence: D = dominant; A = abundant; C = common; U = uncommon; R = rare. Biogeographical affinity: CT = Circumtropical; EP = Eastern Pacific; EP+ATL = Eastern Pacific and Atlantic oceans; EP+IP = Eastern Pacific and Indo-Pacific; MEX = Mexican waters of the Pacific; TEP = Tropical Eastern Pacific. IUCN Categories: EN = Endangered; VU = Vulnerable; DD = Data deficient; NT = Near threatened; LC = Least concern; NE = Not evaluated. - = No data.

CLASS/Order/Family	Species	Record designation	Catalog number of collected specimens	Frequency of occurrence	Bio-geographical affinity	IUCN Categories
**CHONDRICHTHYES**						
**Carcharhiniformes**						
Sphyrnidae	*Sphyrna lewini* (Griffith & Smith, 1834)	R1	-	-	CT	EN
**Rajiformes**						
Narcinidae	*Diplobatis ommata* (Jordan & Gilbert, 1890)	V	-	R	TEP	VU
Rhinobatidae	*Rhinobatos glaucostigma* Jordan & Gilbert, 1883	SIO, R1	SIO 70-238	-	TEP	DD
	*Rhinobatos leucorhynchus* Günther, 1867	V		R	TEP	NT
	*Zapteryx xyster* Jordan & Evermann, 1896	V, SIO	SIO 70-237	R	TEP	DD
**Myliobatiformes**						
Gymnuridae	*Gymnura marmorata* (Cooper, 1864)	R1	-	-	TEP	LC
Myliobatidae	*Aetobatus narinari* (Euphrasen, 1790)	V, R2	-	R	CT	NT
Urotrygonidae	*Urobatis concentricus* Osburn & Nichols, 1916	V, R2	-	R	MEX	DD
	*Urobatis halleri* (Cooper, 1863)	SIO, R1	SIO 70-237	-	TEP	LC
	*Urotrygon munda* Gill, 1863	R1		-	TEP	DD
	*Urotrygon rogersi* (Jordan & Starks, 1895)	SIO	SIO 70-238	-	TEP	DD
**ACTINOPTERYGII**						
**Albuliformes**						
Albulidae	*Albula pacifica* (Beebe, 1942)†	R1	-	-	EP	NE
**Anguilliformes**						
Congridae	*Ariosoma gilberti* (Ogilby, 1898)	SIO	SIO 70-237	-	TEP	LC
	*Paraconger californiensis* Kanazawa, 1961	V, SIO	SIO 70-235	R	TEP	LC
Muraenidae	*Gymnomuraena zebra* (Shaw, 1797)	V, R2	-	R	EP+IP	NE
	*Gymnothorax castaneus* (Jordan & Gilbert, 1883)	V	-	R	TEP	LC
	*Muraena lentiginosa* Jenyns, 1842	V, R2	-	R	TEP	LC
Ophichthidae	*Apterichtus equatorialis* (Myers & Wade, 1941)	C	LEMA-PE138	-	TEP	LC
	*Myrichthys tigrinus* Girard, 1859	V, R1, R2	-	R	TEP	LC
	*Ophichthus triserialis* (Kaup, 1856)	R1	-	-	TEP	LC
	*Quassiremus nothochir* (Gilbert, 1890)	V	-	R	TEP	LC
**Clupeiformes**						
Clupeidae	*Harengula thrissina* (Jordan & Gilbert, 1882)	C, R1	LEMA-PE92	-	EP	LC
	*Lile stolifera* (Jordan & Gilbert, 1882)	R1	-	-	TEP	LC
Pristigasteridae	*Pliosteostoma lutipinnis* (Jordan & Gilbert, 1882)	R1	-	-	TEP	LC
Engraulidae	*Anchoa ischana* (Jordan & Gilbert, 1882)	R1	-	-	TEP	LC
	*Anchoa scofieldi* (Jordan & Culver, 1895)	R1	-	-	TEP	LC
**Aulopiformes**						
Synodontidae	*Synodus evermanni* Jordan & Bollman, 1890	SIO	SIO 70-237	-	TEP	LC
	*Synodus lacertinus* Gilbert, 1890	V, C, R1	LEMA-PE97	R	EP	LC
	*Synodus scituliceps* Jordan & Gilbert, 1882	SIO, R1	SIO 70-237	-	TEP	LC
	*Synodus sechurae* Hildebrand, 1946	SIO	SIO 70-237	-	TEP	LC
**Gadiformes**						
Bregmacerotidae	*Bregmaceros bathymaster* Jordan & Bollman, 1890	SIO	SIO 70-168	-	TEP	LC
**Ophidiiformes**						
Bythitidae	*Ogilbia boydwalkeri* Møller, Schwarzhans & Nielsen, 2005	SIO	SIO 70-165	-	TEP	LC
	*Ogilbia ventralis* (Gill, 1863)	C	LEMA-PE135	-	MEX	LC
**Batrachoidiformes**						
Batrachoididae	*Porichthys ephippiatus* Walker & Rosenblatt, 1988	SIO	SIO 70-168, 235, 237, 238	-	TEP	LC
**Lophiiformes**						
Antennariidae	*Antennatus coccineus* (Lesson, 1831)	C	LEMA-PE70, 71	-	EP+IP	NE
	*Antennatus sanguineus* (Gill, 1863)	C, SIO	LEMA-PE50, 51 SIO 70-167	-	TEP	LC
**Gobiesociformes**						
Gobiesocidae	*Arcos erythrops* (Jordan & Gilbert, 1882)	C, SIO	LEMA-PE74 SIO 70-167	-	MEX	LC
	*Gobiesox adustus* Jordan & Gilbert, 1882	SIO	SIO 70-167	-	TEP	LC
	*Gobiesox papillifer* Gilbert, 1890	C	LEMA-PE95	-	TEP	LC
**Atheriniformes**						
Atherinopsidae	*Atherinella eriarcha* Jordan & Gilbert, 1882	SIO, R1	SIO 70-167	-	TEP	LC
**Beloniformes**						
Belonidae	*Ablennes hians* (Valenciennes, 1846)	C, SIO	LEMA-PE60 SIO 70-166	-	CT	NE
	*Platybelone argalus* (Lesueur, 1821)	R1	-	-	CT	LC
	*Tylosurus fodiator* Jordan & Gilbert, 1882	R1	-		CT	LC
**Beryciformes**						
Holocentridae	*Myripristis leiognathus* Valenciennes, 1846	V, SIO, R2	SIO 70-167	R	TEP	LC
	*Sargocentron suborbitalis* (Gill, 1863)	V, R2	-	U	TEP	LC
**Syngnathiformes**						
Fistulariidae	*Fistularia commersonii* Rüppel, 1838	V, SIO, R2	SIO 70-167	R	EP+IP	NE
Syngnathidae	*Hippocampus ingens* Girard, 1858	C	LEMA-PE99	-	EP	VU
**Scorpaeniformes**						
Scorpaenidae	*Pontinus* sp. 1‡	C	LEMA-PE132	-	-	-
	*Pontinus* sp. 2‡	C	LEMA-PE136	-	-	-
	*Scorpaena mystes* Jordan & Starks, 1895	V, C, R2	LEMA-PE102	R	EP	LC
	*Scorpaena sonorae* Jenkins & Evermann, 1889	SIO	SIO 70-238	-	MEX	LC
	*Scorpaenodes xyris* (Jordan & Gilbert, 1882)	C, SIO	LEMA-PE112, 114, 115 SIO 70-167	-	EP	LC
Triglidae	*Prionotus stephanophrys* Lockington, 1881	SIO	SIO 70-168	-	TEP	LC
**Perciformes**						
Epinephelidae	*Alphestes immaculatus* Breder, 1936	V, R2	-	U	TEP	LC
	*Cephalopholis panamensis* (Steindachner, 1877)	V, R2	-	C	TEP	LC
	*Dermatolepis dermatolepis* (Boulenger, 1895)	V	-	R	EP	LC
	*Epinephelus labriformis* (Jenyns, 1840)	V, SIO, R2	SIO 70-167	D	EP	LC
	*Paranthias colonus* (Valenciennes, 1846)	V	-	R	TEP	LC
	*Rypticus bicolor* Valenciennes, 1846	V, SIO	SIO 70-167	R	TEP	LC
	*Rypticus nigripinnis* Gill, 1861	V	-	R	TEP	LC
Serranidae	*Serranus psittacinus* Valenciennes, 1846	V, SIO, R2	SIO 70-167	U	TEP	LC
Apogonidae	*Apogon pacificus* (Herre, 1935)	V, SIO	SIO 70-167	R	EP	LC
	*Apogon retrosella* (Gill, 1862)	V, SIO, R2	SIO 70-167	R	TEP	LC
Carangidae	*Caranx caballus* Günther, 1868	V, R2	-	R	EP	LC
	*Caranx sexfasciatus* Quoy & Gaimard, 1825	V, R1	-	R	EP+IP	LC
	*Carangoides otrynter* (Jordan & Gilbert, 1883)	C	LEMA-PE56	-	EP	LC
	*Carangoides vinctus* (Jordan & Gilbert, 1882)	R1	-	-	TEP	LC
	*Chloroscombrus orqueta* Jordan & Gilbert, 1883	R1	-	-	EP	LC
	*Gnathanodon speciosus* (Forsskål, 1775)	V, R1	-	R	EP+IP	NE
	*Hemicaranx leucurus* (Günther, 1864)	R1	-	-	TEP	LC
	*Oligoplites saurus* (Bloch & Schneider, 1801)	R1	-	-	TEP	NE
	*Selene brevoortii* (Gill, 1863)	C	LEMA-PE103	-	EP	LC
	*Trachinotus paitensis* Cuvier, 1832	R1	-	-	TEP	LC
	*Trachinotus rhodopus* Gill, 1863	C, R1	LEMA-PE108, 113	-	EP	LC
Lutjanidae	*Hoplopagrus guentherii* Gill, 1862	V, R1	-	R	TEP	LC
	*Lutjanus argentiventris* (Peters, 1869)	V, R1, R2	-	U	TEP	LC
	*Lutjanus colorado* Jordan & Gilbert, 1882	R1	-	-	TEP	LC
	*Lutjanus guttatus* (Steindachner, 1869)	V, R1, R2	-	R	EP	LC
	*Lutjanus inermis* (Peters, 1869)	V	-	R	TEP	LC
	*Lutjanus novemfasciatus* Gill, 1862	V, C, R1, R2	LEMA-PE119, 120	R	TEP	LC
	*Lutjanus viridis* (Valenciennes, 1846)	V, R2	-	R	TEP	LC
Gerreidae	*Diapterus peruvianus* (Cuvier, 1830)	R1	-	-	TEP	LC
	*Eucinostomus dowii* (Gill, 1863)	SIO	SIO 70-237	-	TEP	LC
	*Eucinostomus gracilis* (Gill, 1862)	SIO, R1	SIO 70-237	-	TEP	LC
	*Gerres simillimus* Regan, 1907	V, R1	-	-	TEP	LC
Haemulidae	*Anisotremus taeniatus* Gill, 1861	SIO	SIO 70-167	-	TEP	LC
	*Haemulon flaviguttatum* Gill, 1862	V, SIO, R1, R2	SIO 70-167	U	EP	LC
	*Haemulon maculicauda* (Gill, 1862)	V, SIO, R2	SIO 70-167	U	TEP	LC
	*Haemulon sexfasciatum* Gill, 1862	V, R2	-	R	TEP	LC
	*Haemulon scudderii* Gill, 1862	R1	-	-	TEP	LC
	*Haemulon steindachneri* (Jordan & Gilbert, 1882)	V, R1, R2	-	U	TEP	LC
	*Microlepidotus brevipinnis* (Steindachner, 1869)	V, SIO, R2	SIO 70-167	R	TEP	LC
	*Pomadasys macracanthus* (Günther, 1864)	R1	-	-	TEP	LC
	*Pomadasys panamensis* (Steindachner, 1876)	R1	-	-	TEP	LC
Sciaenidae	*Cynoscion nannus* Castro-Aguirre & Arvizu-Martínez, 1976	SIO	SIO 70-168	-	TEP	LC
	*Pareques fuscovittatus* (Kendall & Radcliffe, 1912)	V, SIO	SIO 70-167	R	MEX	LC
	*Umbrina xanti* Gill, 1862	R1	-	-	TEP	LC
Polynemidae	*Polydactylus approximans* (Lay & Bennett, 1839)	R1	-	-	TEP	LC
Mullidae	*Mulloidichthys dentatus* (Gill, 1862)	V, R2	-	C	TEP	LC
	*Pseudupeneus grandisquamis* (Gill, 1863)	V, R1	-	R	TEP	LC
Kyphosidae	*Kyphosus vaigiensis* (Quoy & Gaimard, 1825)	V, R2	-	R	EP	NE
	*Kyphosus elegans* (Peters, 1869)	V, R1, R2	-	R	TEP	LC
Chaetodontidae	*Chaetodon humeralis* Günther, 1860	V, R1, R2	-	A	EP	LC
	*Johnrandallia nigrirostris* Gill, 1862	V, SIO, R2	SIO 70-167	U	TEP	LC
Pomacanthidae	*Holocanthus passer* Valenciennes, 1846	V, SIO, R2	SIO 70-167	C	TEP	LC
	*Pomacanthus zonipectus* (Gill, 1862)	V, R2	-	R	TEP	LC
Pomacentridae	*Abudefduf declivifrons* (Gill, 1862)	V	-	R	TEP	LC
	*Abudefduf troschelii* (Gill, 1862)	V, R2	-	U	TEP	LC
	*Chromis atrilobata* Gill, 1862	V, SIO, R2	SIO 70-167	U	TEP	LC
	*Microspathodon bairdii* (Gill, 1862)	V, R2	-	R	TEP	LC
	*Microspathodon dorsalis* (Gill, 1862)	V, SIO, R2	SIO 70-167	A	TEP	LC
	*Stegastes acapulcoensis* (Fowler, 1944)	V, R2	-	A	TEP	LC
	*Stegastes flavilatus* (Gill, 1862)	V, SIO, R2	SIO 70-167	D	TEP	LC
	*Stegastes leucorus* (Gilbert, 1892)	V, R2	-	R	MEX	VU
	*Stegastes rectifraenum* (Gill, 1862)	V, R2	-	R	TEP	LC
Cirrhitidae	*Cirrhitichthys oxycephalus* (Bleeker, 1855)	V	-	R	TEP	NE
	*Cirrhitus rivulatus* Valenciennes, 1846	V	-	C	TEP	LC
Mugilidae	*Mugil curema* Valenciennes, 1836	V, R1, R2	-	R	EP+ATL	NE
	*Chaenomugil proboscideus* (Günther, 1861)	R1	-	-	TEP	LC
Labridae	*Bodianus diplotaenia* (Gill, 1862)	V, SIO, R2	SIO 70-167	C	TEP	LC
	*Halichoeres chierchiae* Di Caporiacco, 1948	V, R1, R2	-	C	TEP	LC
	*Halichoeres dispilus* (Günther, 1864)	V, C, SIO, R2	LEMA-PE93, 127, 128 SIO 70-167	D	TEP	LC
	*Halichoeres melanotis* (Gilbert, 1890)	V, R2	-	R	TEP	LC
	*Halichoeres nicholsi* (Jordan & Gilbert, 1882)	V, SIO, R2	SIO 70-167	A	TEP	LC
	*Halichoeres notospilus* (Günther, 1864)	V, R2	-	U	TEP	LC
	*Iniistius pavo* (Valenciennes, 1840)	C	LEMA-PE133	-	EP+IP	LC
	*Novaculichthys taeniourus* (Lacepède, 1801)	V, R2	-	R	EP+IP	LC
	*Thalassoma grammaticum* Gilbert, 1890	V, R2	-	R	EP+IP	LC
	*Thalassoma lucasanum* (Gill, 1862)	V, R2	-	C	TEP	LC
Scaridae	*Nicholsina denticulata* (Evermann & Radcliffe, 1917)	V, R2	-	R	EP	LC
	*Scarus ghobban* Forsskål, 1775	V, R2	-	R	EP+IP	LC
	*Scarus perrico* Jordan & Gilbert, 1882	V, R2	-	R	TEP	LC
Tripterygiidae	*Axoclinus storeyae* (Brock, 1940)	V, C	-	R	MEX	LC
	*Enneanectes carminalis* (Jordan & Gilbert, 1882)	C, SIO	LEMA-PE121 SIO 70-167	-	TEP	LC
	*Enneanectes glendae* Rosenblatt, Miller & Hastings, 2013	V, SIO	SIO 70-167	R	MEX	NE
	*Enneanectes macrops* Rosenblatt, Miller & Hastings, 2013	SIO	SIO 70-167	-	MEX	NE
Dactyloscopidae	*Dactyloscopus amnis* Miller & Briggs, 1962	C	LEMA-PE78	-	TEP	LC
	*Gillellus arenicola* Gilbert, 1890	C	LEMA-PE117	-	TEP	LC
Labrisomidae	*Labrisomus xanti* (Gill, 1860)	V	-	R	MEX	LC
	*Malacoctenus ebisui* Springer, 1959	V, C, SIO, R2	LEMA-PE100, 107 SIO 70-167	R	TEP	LC
	*Malacoctenus mexicanus* Springer, 1959	C, SIO	LEMA-PE98 SIO 70-167	-	TEP	LC
	*Malacoctenus polyporosus* Springer, 1959	V, C	LEMA-PE110	R	TEP	LC
	*Malacoctenus tetranemus* (Cope, 1877)	C, SIO	LEMA-PE96, 109 SIO 70-167	-	TEP	LC
	*Paraclinus tanygnathus* Rosenblatt & Parr, 1969	C	LEMA-PE101, 106, 111	-	MEX	LC
	*Starksia spinipenis* (Al-Uthman, 1960)	V, C, SIO	LEMA-PE118 SIO 70-167	R	MEX	LC
Chaenopsidae	*Acanthemblemaria macrospilus* Brock, 1940	V, C, R2	LEMA-PE87, 134	R	MEX	LC
	*Chaenopsis* sp.§	SIO	SIO 14-41	-	-	-
	*Coralliozetus boehlkei* Stephens, 1963	C	LEMA-PE84,85	-	TEP	LC
	*Ekemblemaria myersi* Stephens, 1963	C, SIO	LEMA-PE80, 86, 104 SIO 70-167	-	TEP	LC
	*Emblemaria piratica* Ginsburg, 1942	C	LEMA-PE81	-	TEP	LC
	*Protemblemaria bicirrus* (Hildebrand, 1946)	C	LEMA-PE90, 105	-	TEP	LC
Blenniidae	*Entomacrodus chiostictus* (Jordan & Gilbert, 1882)	C	LEMA-PE137	-	TEP	LC
	*Hypsoblennius brevipinnis* (Günther, 1861)	C	LEMA-PE89	-	TEP	LC
	*Ophioblennius steindachneri* Jordan & Evermann, 1898	V, SIO, R2	SIO 70-167	U	TEP	LC
	*Plagiotremus azaleus* (Jordan & Bollman, 1890)	V, SIO, R2	SIO 70-167	R	EP	LC
Eleotridae	*Eleotris picta* Kner, 1863	R1	-	-	TEP	LC
	*Gobiomorus maculatus* (Günther, 1859)	R1	-	-	TEP	LC
Gobiidae	*Coryphopterus urospilus* Ginsburg, 1938	V, SIO, R2	LEMA-PE94 SIO 70-167	R	TEP	LC
	*Ctenogobius sagittula* (Günther, 1862)	C	LEMA-PE62	-	EP	LC
	*Elacatinus puncticulatus* (Ginsburg, 1938)	V, C, SIO, R2	LEMA-PE88, 116 SIO 70-167	R	TEP	LC
	*Gymneleotris seminuda* (Günther, 1864)	V	-	R	TEP	LC
	*Tigrigobius digueti* (Pellegrin, 1901)	C, SIO	LEMA-PE69,83 SIO 70-167	R	MEX	NE
Microdesmidae	*Microdesmus dipus* Günther, 1864	C	LEMA-PE66	-	TEP	DD
	*Microdesmus dorsipunctatus* Dawson, 1968	C	LEMA-PE67, 82	-	TEP	DD
Ephippidae	*Chaetodipterus zonatus* (Girard, 1858)	R1	-	-	EP	LC
Zanclidae	*Zanclus cornutus* (Linnaeus, 1758)	V, R2	-	R	EP+IP	NE
Acanthuridae	*Acanthurus xanthopterus* Valenciennes, 1835	V, R1	-	R	EP+IP	LC
	*Prionurus punctatus* Gill, 1862	V, R2	-	R	TEP	LC
Sphyraenidae	*Sphyraena ensis* Jordan & Gilbert, 1882	C, R1	LEMA-PE129	-	TEP	LC
Scombridae	*Euthynnus lineatus* Kishinouye, 1920	V	-	R	EP+IP	LC
**Pleuronectiformes**						
Paralichthyidae	*Cyclopsetta* sp.‡	C	LEMA-PE130	-	-	-
	*Etropus crossotus* Jordan & Gilbert, 1882	R1	-	-	EP+ATL	NE
	*Etropus* sp.‡	C	LEMA-PE123a	-	-	-
	*Syacium latifrons* (Jordan & Gilbert, 1882)	SIO	SIO 70-238	-	TEP	LC
	*Syacium ovale* (Günther, 1864)	SIO	SIO 70-237	-	TEP	LC
	*Syacium* sp.‡	C	LEMA-PE124	-	-	-
Bothidae	*Bothus constellatus* (Jordan, 1889)	SIO	SIO 70-237, 238	-	EP+IP	NE
	*Monolene dubiosa* Garman, 1899	SIO	SIO 70-168	-	TEP	LC
Cynoglossidae	*Symphurus atramentatus* Jordan & Bollman, 1890	SIO	SIO 70-237	-	TEP	LC
	*Symphurus leei* Jordan & Bollman, 1890	C, SIO	LEMA-PE122 SIO 70-235	-	TEP	LC
	*Symphurus melanurus* Clark, 1936	C	LEMA-PE131	-	TEP	LC
	*Symphurus* sp.‡	C	LEMA-PE123b, 125, 126	-	-	-
**Tetraodontiformes**						
Balistidae	*Balistes polylepis* Steindachner, 1876	V, R2	-	R	EP+IP	LC
	*Pseudobalistes naufragium* (Jordan & Starks, 1895)	V, R2	-	R	TEP	LC
	*Sufflamen verres* (Gilbert & Starks, 1904)	V, R2	-	C	TEP	LC
Monacanthidae	*Aluterus scriptus* (Osbeck, 1765)	V, R2	-	R	CT	NE
Ostraciidae	*Cantherhines dumerilii* (Hollard, 1854)	V	-	R	EP+IP	NE
	*Ostracion meleagris* Shaw, 1796	V, R2	-	R	EP+IP	NE
Tetraodontidae	*Arothron hispidus* (Linnaeus, 1758)	V	-	R	EP+IP	NE
	*Arothron meleagris* (Anonymous, 1798)	V, R1, R2	-	U	EP+IP	NE
	*Canthigaster punctatissima* (Günther, 1870)	V, R2	-	R	TEP	LC
	*Sphoeroides annulatus* (Jenyns, 1842)	V, SIO, R1, R2	SIO 70-238	R	EP	LC
	*Sphoeroides lobatus* (Steindachner, 1870)	V, SIO, R2	SIO 70-237, 238	R	TEP	LC
Diodontidae	*Chilomycterus reticulatus* Linnaeus, 1758	V, R2	-	R	CT	NE
	*Diodon holocanthus* Linnaeus, 1758	V, SIO, R2	SIO 70-167	C	CT	NE
	*Diodon hystrix* Linnaeus, 1758	V, R1, R2	-	R	CT	NE

† considered as *Albula nemoptera* by Espinoza-Pérez et al. (2002), designated as *Albula pacifica* by Pfeiler (2008).

‡ These individuals represent juveniles too small to be accurately identified.

§ This individual is an undescribed species previously found in Costa Rica.

The fish species richness of Bahía Chamela (196 species) is greater than in other surveyed MPAs of the Mexican Pacific, including Bahía de Los Ángeles (93 species), Bahía Loreto (66), Cabo Pulmo (62), and Isla Isabel (118) in the Gulf of California (Campos-Dávila et al. 2005, Alvarez-Filip et al. 2006, Galván-Villa et al. 2010, Viesca-Lobaton et al. 2008, Mascareñas-Osorio et al. 2011); Islas Marietas (46) (Solís-Gil and Jiménez-Quiroz 2004) in the central Mexican Pacific; and Bahías de Huatulco (112) in the southern Mexican Pacific (López-Pérez et al. 2010). Only three species (*Hippocampus ingens*, *Holacanthus passer*, *Pomacanthus zonipectus*) occurring in the bay have been designated with special protection category by the Mexican Official Norm 059-ECOL-2010. Furthermore, in the red list of the International Union for Conservation of Nature (IUCN 2015) three species are assessed as vulnerable (*Diplobatis ommata*, *Hippocampus ingens*, *Stegastes leucorus*) and one as endangered (*Sphyrna lewini*) (Table 1).

Fifty-four percent of the species was recorded using visual census. The composition of the fish assemblage of the bay is characterized mainly by rare species (72%). Three species are categorized as dominant: *Epinephelus labriformis*, *Stegastes flavilatus*, and *Halichoeres dispilus*; these species are widely distributed along the Mexican Pacific and are recognized as important in the reef-fish assemblage structure for this bay and other MPAs of the Mexican Pacific because of their high abundance and biomass (Galván-Villa 2015). Another four species are categorized as abundant: *Chaetodon humeralis*, *Microspathodon dorsalis*, *Stegastes acapulcoensis*, and *Halichoeres nicholsi*; nine as common, and 13 as uncommon. The number of species inhabiting the bay may increase after checking additional details of some of the collected specimens and published records. Additions may include undescribed species, juvenile stages from different species, or records from publications with erroneous determinations. For example, a single female individual of chaenopsid pike-blenny (*Chaenopsis* sp.) that was collected from sandy bottom of the bay corresponds to an undescribed species distributed from Mexico to Costa Rica (Hastings *pers obs*). Also three individuals of *Pontinus* (sp. 1 and sp. 2) were collected, but due to their small size (< 2 cm), the identification of species was not possible. They probably correspond to *Pontinus furcirhinus* or *Pontinus sierra*, as both species have been recorded in the area (Robertson and Allen 2015). Another five juvenile individuals of flounders (Paralichthyidae) and eight tonguefishes (Cynoglossidae) collected by the biological dredge from sandy bottoms were not identified to species level. Future careful taxonomic work on these and other specimens would increase the number of species recorded from the bay.

**Figure 2.**
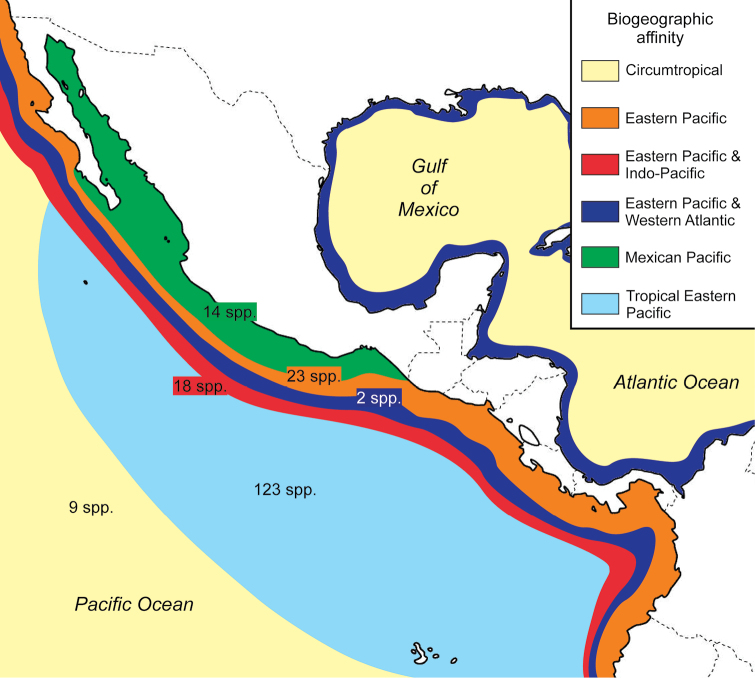
Map showing the number of fish species recorded in Bahía Chamela and their biogeographic affinities.

**Figure 3.**
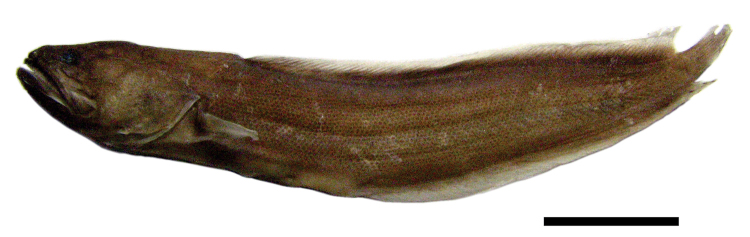
*Ogilbia ventralis*. LEMA-PE135, ♂, 56 mm SL, Pacific Mexico, Bahía Chamela. Scale 10 mm. Photo by CMGV.

*Biogeographic affinity.* Most fish species recorded in Bahía Chamela are widely distributed in the tropical eastern Pacific (123 spp = 66%) (Figure 2). Twenty-three species occur in the eastern Pacific, and 18 occur in both eastern and Indo-Pacific waters. Fourteen species are endemic in Mexican waters of the Pacific. One specimen of the Gulf Brotula, *Ogilbia ventralis*, was collected with clove oil anesthetic from under rocks, depth 6 m at the islet Novillos (Figure 3). This record represents a southern range extension for this species, known previously from the Gulf of California and southern part of the outer Baja peninsula. Bahía Chamela is the type locality for a second *Ogilbia* species, *Ogilbia boydwalkeri* (Møller et al. 2005). The festive drum fish, *Pareques fuscovitattus*, is the only endemic species in the Mexican Province (Robertson and Allen 2015). Nine species are circumtropical, and another two (*Mugil curema* and *Etropus crossotus*) occur in both the eastern Pacific and western Atlantic regions. Seven undetermined or non-described species were excluded from the analysis of biogeographic affinity.

Previous studies considered *Haemulon steindachneri* (Haemulidae) to occur in both eastern Pacific and western Atlantic oceans, although recently molecular analysis found that these two populations belong to different species, so the valid distribution of this nominal species is the TEP (Rocha et al. 2008). Future review of other species that reportedly occur in both oceans is important to define valid distributions. Finally, according to Robertson and Cramer (2009), the fish richness of Bahía Chamela is most similar to that of the Panama biogeographic province, but there is an important contribution of species from the Gulf of California and outer Baja peninsula and a few species from other oceans.
